# Amyloid-β Oligomers Interact with Neurexin and Diminish Neurexin-mediated Excitatory Presynaptic Organization

**DOI:** 10.1038/srep42548

**Published:** 2017-02-13

**Authors:** Yusuke Naito, Yuko Tanabe, Alfred Kihoon Lee, Edith Hamel, Hideto Takahashi

**Affiliations:** 1 Synapse Development and Plasticity Research Unit, Institut de recherches cliniques de Montréal, Montréal, Québec, H2W 1R7, Canada; 2 Integrated Program in Neuroscience, McGill University, Montréal, Québec, H3A 2B4, Canada; 3 Laboratory of Cerebrovascular Research, Montreal Neurological Institute, McGill University, Montréal, Québec, H3A 2B4, Canada; 4 Department of Medicine, Université de Montréal, Montréal, Québec, Canada, H3T 1J4; Division of Experimental Medicine, McGill University, Montréal, Québec, H3A 0G4, Canada

## Abstract

Alzheimer’s disease (AD) is characterized by excessive production and deposition of amyloid-beta (Aβ) proteins as well as synapse dysfunction and loss. While soluble Aβ oligomers (AβOs) have deleterious effects on synapse function and reduce synapse number, the underlying molecular mechanisms are not well understood. Here we screened synaptic organizer proteins for cell-surface interaction with AβOs and identified a novel interaction between neurexins (NRXs) and AβOs. AβOs bind to NRXs via the N-terminal histidine-rich domain (HRD) of β-NRX1/2/3 and alternatively-spliced inserts at splicing site 4 of NRX1/2. In artificial synapse-formation assays, AβOs diminish excitatory presynaptic differentiation induced by NRX-interacting proteins including neuroligin1/2 (NLG1/2) and the leucine-rich repeat transmembrane protein LRRTM2. Although AβOs do not interfere with the binding of NRX1β to NLG1 or LRRTM2, time-lapse imaging revealed that AβO treatment reduces surface expression of NRX1β on axons and that this reduction depends on the NRX1β HRD. In transgenic mice expressing mutated human amyloid precursor protein, synaptic expression of β-NRXs, but not α-NRXs, decreases. Thus our data indicate that AβOs interact with NRXs and that this interaction inhibits NRX-mediated presynaptic differentiation by reducing surface expression of axonal β-NRXs, providing molecular and mechanistic insights into how AβOs lead to synaptic pathology in AD.

Alzheimer’s disease (AD) is characterized by the accumulation of toxic amyloid-β (Aβ)-peptides, the principal constituent of plaques in the brains of AD patients^1^,^2^. In addition, loss of synapses in the brain is an early pathological feature of AD and is the best correlate of cognitive impairment^3^,^4^. Experimentally, Aβ oligomers (AβOs) cause synapse loss and synapse dysfunction both *in vitro* and in mouse models of AD^4^,^5^,^6^. The *in vitro* experiments show that the application of soluble AβOs to hippocampal slices or cultured neurons decreases immunoreactivity for pre- and post-synaptic proteins^7^,^8^,^9^,^10^,^11^,^12^ and the density of dendritic spines^9^,^13^,^14^,^15^,^16^. Further, Aβ treatment of hippocampal slices distorts synaptic plasticity^4^,^5^,^6^,^17^. Specifically, Aβ treatment blocks long-term potentiation (LTP) and enhances long-term depression (LTD). Thus, synapses exhibit vulnerability to Aβ. However, little is known about the molecular mechanisms underlying this vulnerability.

Synaptic organizing complexes are trans-synaptic adhesion molecules with an ability to promote pre- and/or post-synaptic organization (hereinafter synaptogenic activity) and are thought to function as essential molecular signals for synapse formation, maturation, maintenance, and plasticity^18^,^19^,^20^,^21^,^22^. The neuroligin (NLG)-neurexin (NRX) complex is one of the most well-studied synaptic organizing complexes, and mutations in this complex are genetic determinants predisposing to cognitive disorders such as autism and schizophrenia^18^,^19^,^21^. Recent studies have identified several other synaptic organizing complexes including TrkC-PTPσ^23^, Slitrk-PTPδ^24^,^25^, the leucine-rich repeat transmembrane protein (LRRTM) 1/2/3-NRX^26^,^27^,^28^,^29^,^30^, calsyntenin3-α-NRX^31^, GluRδ-cerebellin-NRX^32^,^33^,^34^, NGL3-LAR^35^,^36^, and IL1RAPL1/IL1RacP-PTPδ^37^,^38^,^39^. Thus there are many synaptic organizing proteins, any of which could be targets for Aβ in synapse disruption.

Indeed, there is recent evidence that Aβ pathogenic processes may affect synaptic organizers. Several organizers including NRXs^40^,^41^, leukocyte antigen-related tyrosine phosphatase (LAR)^42^,^43^ and NLG1^44^ are cleaved by proteinases involved in the generation of Aβ, indicating that their function may be altered coordinately with Aβ production. Also, AβOs bind to soluble NLG1 ectodomain^45^. These results emphasize the importance of understanding whether and how AβOs affect the physiological roles of synaptic organizing complexes. But, there has been no published study that has systematically tested for physical and functional interactions of synaptic organizing complex proteins with AβOs.

In this study, we performed cell surface binding assays using soluble AβOs to screen for their interaction with synaptic organizers expressed at the cell surface. We found that AβOs interact with NRX family members and defined their AβO-binding domains. AβOs diminish NRX-mediated presynaptic organization by decreasing β-NRX expression on the axonal surface, although this does not affect NRX-NLG1 interaction or NRX-LRRTM2 interaction. In a transgenic mouse line with increased production of Aβ species, synaptic expression of β-NRXs is decreased. Together, our results indicate that AβOs interact with NRXs and that this interaction disrupts NRX-based synapse organization by destabilizing surface β-NRX on axons.

## Results

### A candidate screen isolates β-neurexins as Aβ oligomer-interacting proteins

**Figure 1:**
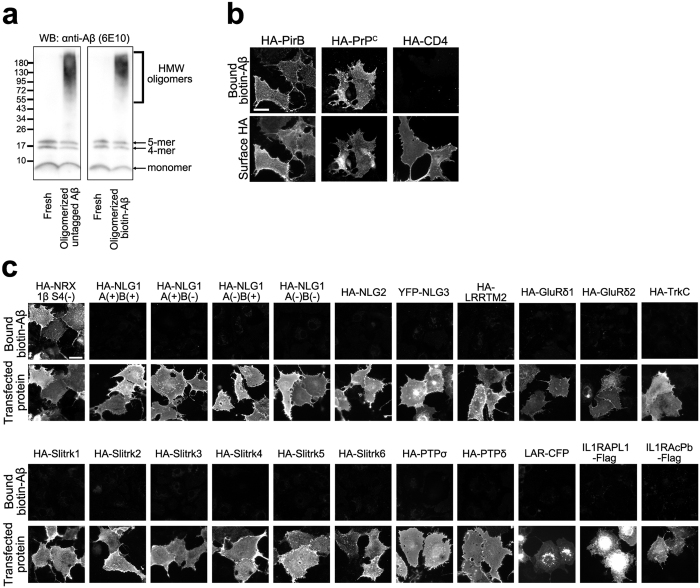
A candidate screen isolates neurexin1β as an Aβ_42_ oligomer-interacting protein. (**a**) Immunoblotting with an anti-β-Amyloid 1–16 antibody (6E10) confirms the formation of soluble oligomers of untagged amyloid-β (1–42) peptide (Aβ) and of biotin-tagged Aβ_42_ peptides (biotin-Aβ). The preparations include both low and high molecular weight (HMW) oligomers. The preparation without an oligomerization incubation step (Fresh) does not include HMW oligomers. Full gel blots for the cropped blots (**a**) are shown in the Supplementary Fig. 4. (**b**) The biotin-Aβ_42_ oligomers bind to COS-7 cells expressing the N-terminal extracellular HA-tagged known Aβ_42_ oligomer receptors, paired immunoglobulin-like receptor B (HA-PirB) and prion protein (HA-PrP^c^), but not those expressing HA-CD4 as a negative control. Surface HA was immunostained to verify expression of these constructs on the COS-7 cell surface. (**c**) Representative images showing cell surface binding assays testing for interaction between biotin-Aβ_42_ oligomers (250 nM, monomer equivalent) and known synaptic organizers. Biotin-Aβ_42_ oligomers were added to COS-7 cells expressing the indicated construct. Note that biotin-Aβ_42_ oligomers bind to COS-7 cells expressing HA-neurexin (NRX)1βS4(−), but not to those expressing any of the other organizers including HA-neuroligin1 (HA-NLG1). For the N-terminal extracellular HA-tagged constructs, surface HA was immunostained to verify expression of the construct on the COS-7 cell surface. Scale bars represent 30 μm (**b,c**).

To test whether there are any synaptic organizers that interact with Aβ oligomers, we performed cell surface binding assays in which soluble oligomers of amyloid-β (1–42) peptide conjugated with biotin (biotin–Aβ_42_) were added onto COS-7 cells expressing each synaptic organizer. We first confirmed that the biotin–Aβ_42_ peptides were properly oligomerized into low and high molecular weight oligomers by western blot analysis (Fig. 1a) and also that the biotin–Aβ_42_ oligomers bound COS-7 cells expressing the known Aβ_42_ oligomer receptors, the paired immunoglobulin-like receptor B (PirB)^46^ and the cellular prion protein (PrP ^c^)^47^ (Fig. 1b). We screened a total of 22 synaptic organizers. We found no significant binding of biotin–Aβ_42_ oligomers on COS-7 cells expressing NLG1 or NLG2 (Fig. 1c) although a previous study reported an interaction between AβOs and NLG1^45^. Instead, we detected significant binding of biotin–Aβ_42_ oligomers on COS-7 cells expressing (NRX1βS4(−)) (Fig. 1c). Interestingly, we did not detect any binding signals on COS-7 cells expressing any of the other synaptic organizers that we tested including type IIa receptor-type protein tyrosine phosphatases (RPTPs: PTPσ, PTPδ, and LAR), LRRTM2, TrkC, and Slitrk family members (Fig. 1c).

**Figure 2:**
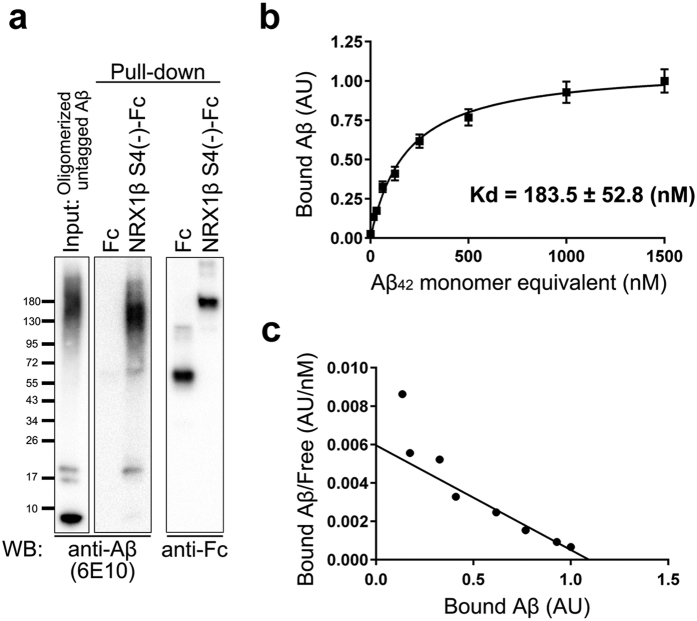
Binding analysis shows that Aβ_42_ oligomers bind neurexin1β in the nanomolar range. (**a**) Pull-down assay of untagged Aβ_42_ oligomers with purified NRX1βS4(−)-Fc proteins. Full gel blots for the cropped blots (**a**) are shown in the Supplementary Fig. 4. (**b**) Saturable binding of biotin-Aβ_42_ oligomers to COS-7 cells expressing HA-NRX1βS4(−). Data are presented as mean ± SEM. (**c**) Scatchard plot of binding data shown in (**b)**. The Kd = 183.5 nM monomer equivalent. (*n* = 30 cells for each plot).

We next checked for a biochemical interaction between NRX1β and AβOs in a pull-down assay using untagged Aβ_42_ oligomers. High-molecular weight Aβ_42_ oligomers and 5-mer Aβ_42_ oligomers, but not Aβ_42_ monomers, were coprecipitated with purified NRX1βS4(−)-Fc proteins pre-immobilized on Protein G magnetic beads (Fig. 2a). In contrast, either untagged Aβ_42_ oligomers or monomers were not coprecipitated with purified Fc proteins pre-immobilized on Protein G magnetic beads. These results suggest that AβOs can interact directly with the extracellular domain of NRX1βS4(−). We next determined the binding affinity by saturation analysis in cell-surface binding assays (Fig. 2b,c). The binding of biotin–Aβ_42_ oligomers to NRX1βS4(−) increased and became saturated with increasing amounts of biotin–Aβ_42_ oligomers. A Scatchard plot of the binding data revealed that the apparent dissociation constant (Kd) value is 183.5 nM monomer equivalent. Thus the interaction between NRX1β and biotin–Aβ_42_ oligomers is within the typical nanomolar range for biologically significant ligand-receptor interactions. Together, these results indicate that Aβ oligomers bind directly with nanomolar affinity to NRX1β.

### Neurexins interact with Aβ oligomers via the N-terminal histidine-rich domain of β-neurexin1/2/3 and an insert at alternative-splicing site 4 of α/β-neurexin1/2

**Figure 3:**
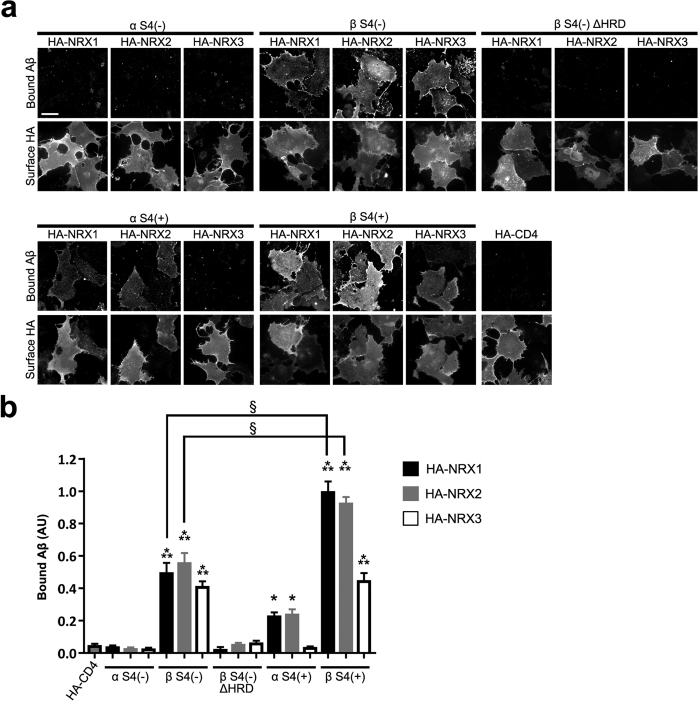
The N-terminal histidine-rich region of β-neurexin1/2/3 and S4 inserts of α/β-neurexin1/2 are responsible for Aβ_42_ oligomer interaction. (**a**) Representative images showing the binding of biotin-Aβ_42_ oligomers (250 nM, monomer equivalent) to COS-7 cells expressing the indicated isoform of extracellularly HA-tagged NRX. S4(−) and S4(+) indicate without and with an insert at splicing site 4, respectively. HA fluorescent signals correspond to surface HA. Note that in assays with S4-negative isoforms, there is no bound biotin-Aβ_42_ oligomer signal on cells expressing α-NRX1, 2, or 3 whereas cells expressing β-NRX1, 2, and 3 all have significant bound biotin-Aβ_42_ oligomer signal. Cells expressing β-NRX1, 2, and 3 lacking the histidine-rich domain (∆HRD) have no biotin-Aβ_42_ oligomer binding signal. Cells expressing S4-positive isoforms of α-NRX1 or 2 or β-NRX1, 2, or 3 have significant bound biotin-Aβ_42_ oligomers. Scale bar represents 30 μm. (**b**) Quantification of bound biotin-Aβ_42_ oligomers for each NRX construct. *n* = 30 cells for each construct from three independent experiments, one-way ANOVA, *P* < 0001. *P < 0.01 and ***P < 0.0001 compared with HA-CD4 and ^§^*P* < 0.0001 between S4(−) and S4(+) by Bonferroni multiple comparisons tests. Data are presented as mean ± SEM.

Given that the NRX family is composed of many different isoforms such as α/β-isoforms and alternative splicing site 4 (S4)-positive or S4-negative isoforms^18^, which have differing binding affinity and selectivity for NRX-interacting proteins, we next tested which NRX isoforms interact with AβOs (Fig. 3). In the case of S4-negative isoforms, biotin–Aβ_42_ oligomers interacted with NRX1β, 2β and 3β at a similar level but not with NRX1α, 2α or 3α, indicating that Aβ_42_ oligomers interact with β-NRX-specific domains in the absence of an insert at S4. Given that the N-terminal histidine-rich domain (HRD; amino acids 50–83 in NRX1β) is unique to the β-isoforms^48^, we next tested the binding of biotin–Aβ_42_ oligomers to COS-7 cells expressing NRX1β lacking the HRD (HA-NRX1β∆HRD) and detected no binding (Fig. 3a,b). Further, COS-7 cells expressing HA-NRX2β∆HRD or HA-NRX3β∆HRD also displayed no binding signal (Fig. 3a,b). These results indicate that the HRD of β-NRX1/2/3 is one of the domains responsible for Aβ_42_ oligomer binding. Next, we investigated Aβ_42_ oligomer-binding to S4-positive NRX isoforms. Biotin–Aβ_42_ oligomers interacted with S4-positive NRX1α and 2α but not 3α (Fig. 3a,b), indicating that the inserts at the S4 site of NRX1 and NRX2 interact with Aβ_42_ oligomers. Indeed, S4-positive NRX1β and 2β displayed stronger binding of Aβ_42_ oligomers than S4-negative NRX1β and 2β, respectively (Fig. 3a,b). The enhancement of binding by the S4 insert is similar to the difference in the binding signals of S4-negative and S4-positive NRX1α and 2α (Fig. 3b), suggesting that Aβ_42_ oligomer-binding to the S4 insert additively increases the binding of Aβ_42_ to NRX1β and 2β. Together, these data indicate that the HRDs of NRX1β, 2β and 3β and the S4 inserts of NRX1α/β and 2α/β are responsible for Aβ_42_ oligomer interaction.

### Aβ treatment diminishes neurexin-mediated presynaptic differentiation

**Figure 4:**
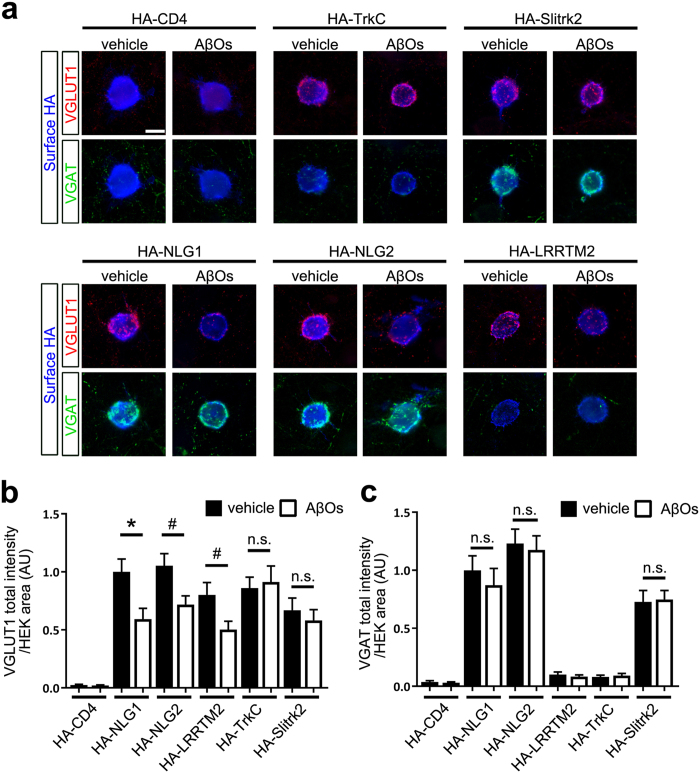
Aβ_42_ oligomers diminish neurexin-mediated excitatory presynaptic differentiation. (**a**) Representative images of triple immunolabeling for VGLUT1, VGAT and surface HA in HEK293 cells expressing the indicated extracellularly HA-tagged construct cocultured with cultured hippocampal neurons and treated with Aβ_42_ oligomers (AβOs, 500 nM, monomer equivalent) or vehicle. HA-CD4 is used as a negative control protein as it lacks synaptogenic activity. AβOs seem not to affect VGLUT1 accumulation induced by an HA-TrkC non-catalytic isoform (HA-TrkC) or VGLUT1 or VGAT accumulation induced by HA-Slitrk2. In contrast, AβOs seem to decrease VGLUT1 accumulation induced by HA-NLG1, HA-NLG2, or HA-LRRTM2. AβOs seem not to affect VGAT accumulation induced by HA-NLG1 or HA-NLG2. Scale bar represents 20 μm. (**b,c**) Quantification of the total intensity of VGLUT1 (**b**) and VGAT (**c**) puncta on HEK293 cells expressing the indicated HA-tagged proteins divided by HEK293 cell area. *n* = 30 cells for each construct from three independent experiments, one-way ANOVA, *P* < 0.0001. ^#^*P* < 0.05 and *P < 0.01 for the indicated comparisons between vehicle control and AβO treatment by Bonferroni multiple comparisons tests. n.s., not significant. Data are presented as mean ± SEM.

NRXs mediate the presynaptic induction activity of NLGs and LRRTM1/2/3 in hippocampal neurons^18^,^19^,^20^,^21^,^22^,^27^,^28^,^29^,^30^. We thus tested whether Aβ_42_ oligomers affect NRX-mediated presynaptic differentiation in coculture-based artificial synapse formation assays (Fig. 4). As reported previously, HEK293 (hereinafter HEK) cells expressing NLG1, NLG2, or LRRTM2 induced the accumulation of the excitatory presynaptic marker VGLUT1. NLG1- or NLG2-expressing HEK cells further induced the accumulation of the inhibitory presynaptic marker VGAT in cocultured hippocampal neurons. Treatment with Aβ_42_ oligomers significantly decreased VGLUT1 accumulation induced by NLG1, NLG2, or LRRTM2 (Fig. 4a,b). Interestingly, treatment with Aβ_42_ oligomers had no effect on VGAT accumulation induced by NLG1 or NLG2 (Fig. 4a,c). These data indicate that Aβ treatment diminishes excitatory, but not inhibitory, presynaptic differentiation induced by NRX-interacting synaptic organizers. Notably, treatment with Aβ_42_ oligomers did not affect TrkC-induced or Slitrk2-induced VGLUT1 accumulation (Fig. 4a,b), which is mediated by RPTPs in cocultured hippocampal neurons^22^,^23^,^24^,^25^, indicating that Aβ treatment diminishes NRX-mediated, but not RPTP-mediated, presynaptic differentiation. Aβ_42_ oligomers did not affect VGAT accumulation induced by HEK cells expressing Slitrk2 (Fig. 4a,c). The observed phenotypes induced by Aβ_42_ oligomers are not due to the reduction of surface expression levels of the tested organizers on HEK cells because Aβ_42_ oligomers did not alter their surface expression (Supplementary Fig. 1). Together these results indicate that Aβ_42_ oligomers selectively diminish NRX-mediated excitatory presynaptic differentiation and also that RPTP-mediated presynaptic differentiation is insensitive to Aβ_42_ oligomers.

### Aβ treatment has no effect on neurexin interaction with neuroligin1 or LRRTM2

**Figure 5:**
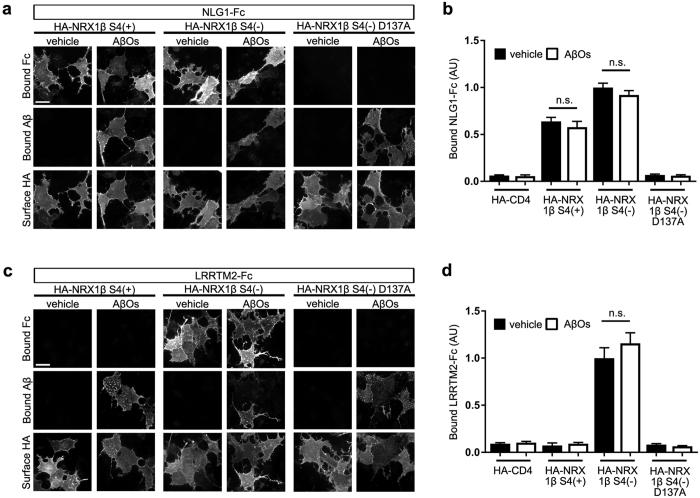
Aβ_42_ oligomers have no effect on the interaction of neurexin1β with neuroligin1 or LRRTM2. (**a,c**) Representative images of triple labeling for bound Fc proteins, bound Aβ_42_ peptides and surface HA on COS-7 cells expressing the indicated extracellularly HA-tagged neurexin (NRX)1β construct. Recombinant neuroligin1-Fc (NLG1-Fc; 20 nM) binds to COS-7 cells expressing HA-NRX1βS4(+) or HA-NRX1βS4(−) but not to those expressing HA-NRX1βS4(−)D137A (**a**), and recombinant LRRTM2-Fc (20 nM) binds to COS-7 cells expressing HA-NRX1βS4(−) but not to those expressing HA-NRX1βS4(+) or HA-NRX1βS4(−)D137A (**c**). Treatment with Aβ_42_ oligomers (AβOs, 500 nM, monomer equivalent) does not seem to affect the binding in any of these cases. Scale bars represent 30 μm. (**b,d**) Quantification of recombinant NLG1-Fc (**b**) or LRRTM2-Fc (**d**) bound to COS-7 cells expressing the indicated HA-NRX1β constructs treated with vehicle control or 500 nM Aβ_42_ oligomers. *n* = 30 cells for each condition from three independent experiments, one-way ANOVA, *P* < 0.0001. n.s., not significant by Bonferroni multiple comparisons test. Data are presented as mean ± SEM.

We next investigated cellular mechanisms by which Aβ_42_ oligomers diminish NRX-mediated presynaptic differentiation. One possibility could be that Aβ_42_ oligomers interfere with the interaction of NRX with NLG1, NLG2, and/or LRRTM2. To test this, we performed cell surface binding assays using recombinant Fc-tagged NLG1 or LRRTM2 ectodomain proteins (NLG1-Fc or LRRTM2-Fc, respectively). Consistent with previous studies, NLG1-Fc bound to COS-7 cells expressing NRX1βS4(−) or (NRX1βS4(+)) (Fig. 5a,b) but not to those expressing a form with a point mutation that completely abolishes NLG interaction, NRX1βS4(−)D137A^49^ (Fig. 5a,b). LRRTM2-Fc bound to COS-7 cells expressing NRX1βS4(−), but not NRX1βS4(+) or NRX1βS4(−)D137A, as reported previously^29^ (Fig. 5c,d). Interestingly, the application of biotin–Aβ_42_ oligomers had no significant effect on the binding of NLG1-Fc or LRRTM2-Fc to any of the NRX1β constructs (Fig. 5b,d). These data indicate that the diminishment of NRX-mediated presynaptic differentiation by Aβ_42_ oligomers is not due to Aβ interference in the interaction of NRXs with NLG1, NLG2, or LRRTM2.

### Aβ treatment decreases surface expression of neurexin1β on axons

**Figure 6:**
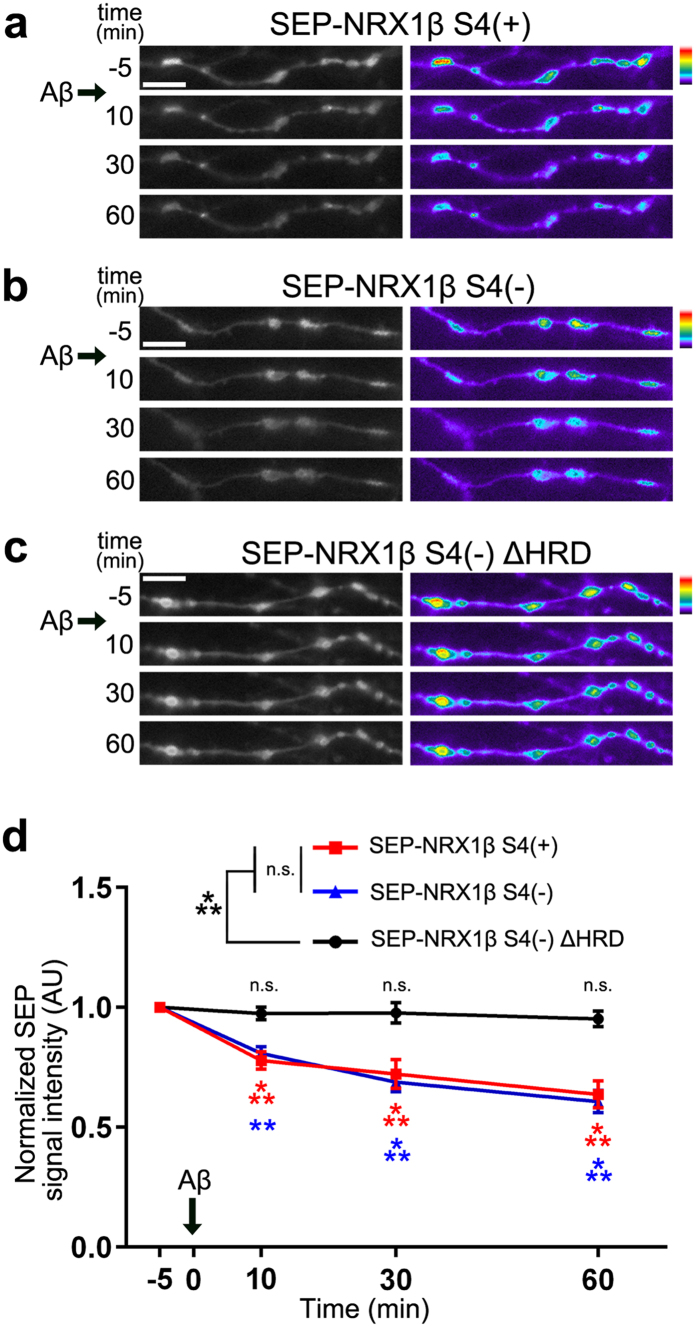
Aβ_42_ oligomers reduce surface expression of neurexin1β on axons. (**a–c**) Representative time-lapse images of axons expressing extracellularly super-ecliptic pHluorin (SEP)-tagged NRX1βS4(+) (**a**), SEP-NRX1βS4(−) (**b**) and SEP-NRX1βS4(−) lacking the histidine-rich domain (SEP-NRX1βS4(−)∆HRD) (**c**) treated with Aβ_42_ oligomers (500 nM, monomer equivalent) at t = 0 min. Scale bars represent 5 μm. (**d**) Quantification of SEP intensity signals at 5 min before and 10, 30 and 60 min after Aβ_42_ oligomer treatment. *n* = 29 for SEP-NRX1βS4(+), *n* = 26 for SEP-NRX1βS4(−), and *n* = 33 for SEP-NRX1βS4(−)ΔHRD puncta from 9 cells for each condition from three independent experiments, two-way repeated measures ANOVA, *F*(3, 255) = 42.94, *P* < 0.0001 for time and *F*(2, 85) = 18.95, *P* < 0.0001 for construct. **P < 0.001, and ***P < 0.0001 compared with SEP signal at 5 min before the treatment, and ***P < 0.0001 compared with SEP-NRX1βS4(−)ΔHRD by Bonferroni multiple comparisons tests. n.s., not significant. Data are presented as mean ± SEM.

We next tested another possible mechanism: Aβ_42_ oligomers could decrease the surface expression of NRXs on axons and thereby diminish NRX-mediated presynaptic differentiation. We cotransfected hippocampal neurons with mCherry (for imaging neuronal morphology) and NRX1β extracellularly tagged with super-ecliptic pHluorin (SEP) (SEP-NRX1β), and then performed time-lapse imaging of SEP-NRX1β expressed on mCherry-positive axons (Fig. 6). SEP is a pH-sensitive GFP variant that yields fluorescence at neutral pH (e.g. on the cell surface) but is quenched at low pH (e.g. inside cytoplasmic vesicles), thus it allows for monitoring the surface expression level of tagged proteins^50^. In mCherry-expressing axons, cotransfected SEP-NRX1βS4(+), SEP-NRX1βS4(−), or SEP-NRX1βS4(−) lacking the HRD (SEP-NRX1βS4(−)∆HRD) had punctate distributions (Fig. 6a–c). Our immunocytochemistry further confirmed that these SEP-NRX1β puncta colocalized with VGLUT1 puncta (Supplementary Fig. 2), suggesting that the SEP-NRX1β proteins likely accumulated at presynaptic boutons. The application of Aβ_42_ oligomers into the extracellular solution decreased the SEP fluorescent signal of SEP-NRX1βS4(+) and SEP-NRX1βS4(−) with similar decay (Fig. 6d). The similarity of the decay suggests that the binding of Aβ_42_ oligomers to the S4 insert is not essential for AβO-induced reduction of NRX surface expression. On the other hand, the application of Aβ_42_ oligomers did not affect the SEP signal of SEP-NRX1βS4(−)∆HRD (Fig. 6d), which does not bind Aβ_42_ oligomers (Supplementary Fig. 3). Thus, the binding of Aβ_42_ oligomers to the HRD is essential for AβO-induced reduction of NRX surface expression. Together with the results of our coculture assays (Fig. 4), these data suggest that Aβ_42_ oligomers diminish NRX-mediated presynaptic differentiation by decreasing surface expression of β-NRXs on axons.

### Synaptic expression of β-neurexin is decreased in a mouse model of Alzheimer’s disease

**Figure 7:**
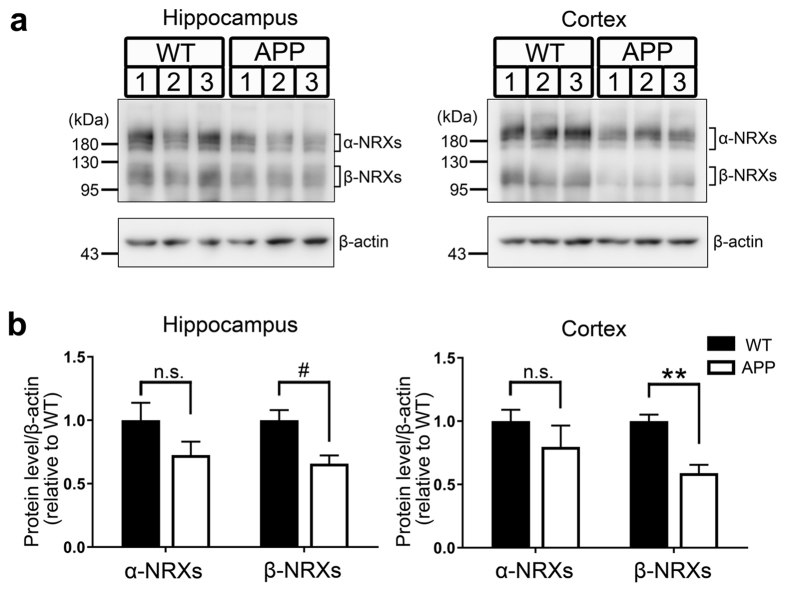
Synaptic expression of endogenous β-neurexins is decreased in J20 APP mice. (**a**) Representative immunoblots of neurexins (NRXs) in synaptosomes from the hippocampus and from the cerebral cortex of J20 APP mice and wild-type (WT) littermates at 6 months of age. The labels 1, 2, and 3 indicate samples from different mice. Full gel blots for the cropped blots (**a**) are shown in the Supplementary Fig. 4. (**b**) Quantification of synaptic expression of β-NRXs (bands indicated by a lower right square bracket in (**a**) and α-NRXs (bands indicated by upper right square bracket in (**a**) normalized to β-actin protein expression in synaptosomes from the hippocampus and the cortex, expressed relative to WT. *n* = 5 samples per genotype for hippocampus, with each n representing pooled hippocampi from two mice. *n* = 6 samples per genotype for cortex, with each *n* representing a cortex from one mouse. Unpaired t tests, ^#^*P* < 0.05 for β-NRXs and *P* = 0.15 for α-NRXs in hippocampus, and **P < 0.001 for β-NRXs and *P* = 0.31 for α-NRXs in cortex. n.s., not significant. Data are presented as mean ± SEM.

Finally, we tested whether AβOs affect synaptic expression of endogenous NRXs *in vivo* (Fig. 7). We prepared synaptosomal fractions from the hippocampus and the cortex of J20 APP mice, transgenic mice expressing a mutant form of human amyloid precursor protein (APP) that have progressively increasing AβO expression and amyloid deposition^51^. When compared to those of wild-type littermates, the hippocampal and cortical synaptosomes from J20 APP mice both had significantly reduced levels of β-NRX proteins, but not α-NRX proteins (Fig. 7a,b). These data indicate that there is selective reduction of endogenous β-NRXs in synapses in the J20 transgenic AD mouse model.

## Discussion

In this study, we uncovered a direct interaction of Aβ_42_ oligomers with NRXs. We further determined that the HRDs of NRX1β, 2β and 3β and S4 inserts of NRX1 and NRX2 are responsible for the interaction between Aβ_42_ oligomers and NRXs. Aβ_42_ oligomers diminish NRX-mediated presynaptic organization by decreasing surface expression of β-NRXs on axons. Further, synaptic expression of endogenous β-NRXs is selectively decreased in a line of transgenic mice with increased production of Aβ peptides. Together, our findings demonstrate that NRX is an interactor of Aβ_42_ oligomers and plays a role in AβO-induced synapse pathology.

It has been well known that AβOs have postsynaptic adverse effects such as the inhibition of LTP, the enhancement of LTD and the loss of dendritic spines (see the review of refs 4 and 6 and references therein). These effects are mediated by Aβ-interacting postsynaptic membrane proteins such as prion^47^,^52^,^53^, PirB^46^ and EphB2^54^, whose binding to Aβ alters the function of postsynaptic NMDA-type glutamate receptor and/or metabotropic glutamate receptor 5 and consequently affects synaptic plasticity and dendritic spine density. However, Aβ accumulates at presynaptic terminals as well as at postsynaptic sites, and Aβ can also distort presynaptic structure and function^6^,^9^,^11^. The mechanisms underlying the presynaptic actions of Aβ are not well understood. NRXs are presynaptic cell-adhesion molecules crucial for presynaptic organization and functions^18^,^19^. Thus the most significant finding of this study is the identification of NRXs as novel AβO-interacting presynaptic membrane proteins. Our data show that Aβ_42_ oligomers interact with NRXs and that this interaction leads to a decrease in NRX expression on the axon surface. NRXs regulate synapse organization through interacting with multiple postsynaptic adhesion molecules including NLG1-4^18^,^19^, LRRTM1/2/3^26^,^27^,^28^,^29^,^30^, and calsyntenin-3^31^. Thus, the NRX family serves as a presynaptic molecular hub to integrate and/or coordinate multiple trans-synaptic organizing signals^22^. Our findings therefore suggest that Aβ_42_ oligomers may dampen the presynaptic hub function of NRXs and thereby disrupt the balance of the multiple synaptic organizing complexes. The dysregulation of NRXs by Aβ_42_ oligomers would therefore be an important mechanism underlying Aβ vulnerability of synapses.

Like members of the NRX family, RPTP family members including PTPσ, PTPδ, and LAR act as presynaptic hubs by mediating trans-synaptic interactions with multiple organizers such as TrkC, Slitrks, NGL-3, and IL1RAPL1^22^. Our binding screen shows that RPTP family members do not interact with Aβ_42_ oligomers. Further, our coculture data suggest that Aβ_42_ oligomers diminish NRX-mediated, but not RPTP-mediated, presynaptic differentiation. Thus, NRX-based synaptic organizing complexes are sensitive to Aβ_42_ oligomers whereas RPTP-based complexes are not. Although synapses are vulnerable to Aβ, the majority of synapses remain after the treatment of cultured neurons with AβOs^13^,^15^ and even at the late stage of AD^3^,^55^. This suggests that synapses exhibit two conflicting properties: Aβ vulnerability and tolerance. Thus, Aβ vulnerability and tolerance of synapses may be partly determined by two different presynaptic hubs: an Aβ-sensitive hub (NRX) and an Aβ-insensitive hub (RPTP).

In our coculture assays, Aβ_42_ oligomers suppress excitatory presynaptic differentiation induced by HEK cells expressing multiple NRX-interacting synaptic organizer proteins: NLG1, NLG2, and LRRTM2. Our results demonstrating disruption of the hub protein NRX represent one mechanism underlying this effect. Direct interaction of AβOs with these NRX interactors could be another possible mechanism. A previous study reported a direct interaction between Aβ_42_ peptides and the NLG1 ectodomain^45^. However, our cell surface binding assay showed no binding of Aβ_42_ oligomers to NLG1-expressing fibroblasts. This discrepancy could result from a difference in the production of the target proteins: the previous study used soluble recombinant NLG1 ectodomain proteins whereas we used membrane-bound NLG1 expressed on the cell surface. The same previous study^45^ and our binding assay show that NLG2 does not interact with Aβ_42_ oligomers and we also show that they do not bind LRRTM2 in cell surface binding assays. These data support the idea that the molecular mechanism by which Aβ_42_ oligomers suppress the synaptogenic activity of NLG1 and other synaptogenic NRX interactors is by binding and decreasing NRXs on the axon surface, rather than by directly binding multiple synaptogenic NRX interactors.

The effects of AβOs may not be limited to synapse organization but may also impact on synaptic function as NRX proteins can regulate neurotransmitter release: α-NRX isoforms regulate Ca^2+^-triggered neurotransmitter release by functionally coupling calcium channels to presynaptic machinery^56^ whereas β-NRX isoforms regulate endocannabinoid-dependent glutamate release probability^57^. A previous study has shown that Aβ increases the presynaptic release probability of glutamate^58^. Thus, the interaction between NRXs and Aβ_42_ oligomers that we have uncovered here could mediate Aβ-dependent glutamate release by changing calcium channel activation and/or endocannabinoid signaling pathways. Future studies using α-NRX1/2 double knockout mice and/or β-NRX1/2/3 triple knockout mice could test this possibility.

Aβ seems to selectively affect glutamatergic (excitatory) presynaptic terminals. A previous study showed that Aβ has no effect on GABA release probability^58^ and our coculture data show that Aβ_42_ oligomers diminish excitatory, but not inhibitory, presynaptic differentiation induced by NLG1/2. Since our data has also shown that NRX is central to Aβ vulnerability, the mechanism underlying differential sensitivity of glutamatergic and GABAergic axons to Aβ likely involves NRX. Our binding assays show that the binding of Aβ_42_ oligomers to NRXs depends on the isoform type (α versus β) and S4 site insertion, and a recent study using single-cell mRNA profiling has shown that there is cell-type-specific expression of NRX isoforms^59^. Thus, comparison of the expression profiles of α- and β-isoforms and S4 splicing in glutamatergic and GABAergic neurons could yield insight into the differing Aβ sensitivity of their axons.

We have shown here that the molecular mechanism of the Aβ-NRX interaction involves two domains: the S4 inserts of NRX 1/2 and the HRDs of β-NRX forms. NRX S4 inserts are critical for determining the binding affinity of NLGs with NRXs and the binding selectivity of LRRTMs with NRXs^18^,^19^,^29^. Although Aβ_42_ oligomers bind to the S4 inserts of NRX1 and NRX2, Aβ_42_ oligomers have no effect on the binding of NLG1 or LRRTM2 to NRX1βS4(+) and also have similar effects on surface expression of SEP-NRX1βS4(+) and SEP-NRX1βS4(−). These findings suggest that the binding of Aβ_42_ oligomers to the S4 insert is not essential for AβO-induced diminishment of NRX-mediated presynaptic differentiation. Instead, our binding assays show that the HRDs of NRX1β, 2β, and 3β are necessary for the binding of Aβ_42_ oligomers to β-NRXs and our time-lapse imaging study demonstrates that the HRD of β-NRXs is crucial for AβO-induced reduction of NRX surface expression on axons. The importance of the HRD for axonal expression of NRX is additionally supported by our *in vivo* finding that β-NRXs (which possess an HRD), but not α-NRXs (which lack HRDs), are decreased significantly in synaptosomes of J20 APP mice. Therefore, the HRD likely contributes to the stabilization of surface β-NRXs on axons under normal physiological conditions.

Our binding assays demonstrate that β-NRX HRDs and NRX1/2 S4 inserts are the domains responsible for Aβ_42_ oligomer binding of NRXs. This is helpful to develop new therapeutic strategies aimed at preventing Aβ-induced synapse pathology, in particular presynaptic dysfunction since NRXs function presynaptically^18^,^56^,^57^. For example, neutralizing antibodies and/or small peptides that block NRX-AβO interactions could normalize presynaptic glutamate release distorted by AβOs. Thus, our findings provide new molecular insights into how Aβ-induced synapse pathology could be prevented and/or reduced.

## Materials and Methods

### Plasmids

To generate a series of extracellularly HA-tagged neurexin (HA-NRX) constructs, cDNA encoding the mature form of each NRX isoform was subcloned into spNRX1β-HA-C1, a vector containing a CMV promoter upstream of the N-terminal signal peptide sequence of NRX1β (spNRX1β) followed by HA and a multiple cloning site. The following NRX vectors were used as a PCR template for the subcloning: intracellular CFP-tagged mouse NRX1βS4(+), 1βS4(−), 1αS4(+), 1αS4(−), 2αS4(+), 2αS4(−), 3αS4(+), and 3αS4(−) (kindly provided by Dr. Ann Marie Craig (University of British Columbia)) and intracellular V5-tagged mouse NRX2βS4(+), 2βS4(−), 3βS4(+), and 3βS4(−) (kindly provided by Dr. Takeshi Uemura (Shinshu University)). For β-NRX constructs lacking their N-terminal histidine-rich domain (HRD), the coding sequence for the mature forms of NRX1β lacking HRD (aa 50–83), NRX2β lacking HRD (aa 54–87) and NRX3β lacking HRD (aa 48–81) were subcloned into spNRX1β-HA-C1 following the NRX1β signal sequence and HA. For extracellularly super-ecliptic pHluorin (SEP)-tagged neurexin1β (SEP-NRX1β) constructs, the coding sequence for the mature form of each NRX1β was subcloned into spNRX1β-SEP-C1, a vector containing a CMV promoter upstream of spNRX1β followed by the SEP coding region and a multiple cloning site. All constructs were verified by DNA sequencing. Further details are described in Supplementary Methods.

### Animals

All animal experiments were carried out in accordance with the Canadian Council on Animal Care guidelines and approved by the IRCM Animal Care Committee and the McGill University Animal Care Committee. We used heterozygous transgenic adult C57BL/6 mice (6 months old, mixed sex) expressing the human amyloid precursor protein (hAPP) carrying the Swedish (K670N, M671L) and Indiana (V717F) familial AD mutations driven by the platelet-derived growth factor (PDGF) β-chain promoter (APP mice, J20 line)^51^ and age-matched wild-type (WT) littermates.

### Preparation of Aβ_42_ oligomers

Aβ(1–42) (r-peptide, A-1002–2, 1 mg) and biotin-tagged Aβ(1–42) (Anaspec, AS-23523-05, 0.5 mg) were used to generate oligomeric forms essentially as described previously^60^. Full details of the Aβ_42_ oligomer preparation are provided in Supplementary Methods. The preparations were stored at −80 °C or used in experiments immediately. Individual Aβ oligomer stocks were never thawed and re-frozen. To confirm oligomer formation, the preparation was run on a 4–20% TGX precast gel (Biorad) and immunoblotted with anti-β-Amyloid 1–16 (1:5000; mouse IgG1; clone 6E10; Covance).

### Neuron culture, coculture-based artificial synapse formation assay and immunocytochemistry

Cultures of rat hippocampal neurons, COS-7 cells, HEK293 cells, coculture-based artificial synapse formation assays, and immunocytochemistry were performed essentially as reported previously^23^,^24^. Transfections into COS-7 and HEK293 cells were performed using TransIT-LT1 (Mirus Bio. LLC). For transfections into hippocampal neurons, the ProFection Mammalian Transfection System (Promega) was used. For artificial synapse formation assays, transfected HEK293 cells were co-cultured with rat hippocampal neurons. Cultures were fixed with parafix solution (4% paraformaldehyde and 4% sucrose in PBS (pH 7.4)) for 12 minutes followed by permeabilization with PBST (PBS + 0.2% Triton X-100). They were incubated with blocking solution (PBS + 3% bovine serum albumin (BSA) and 5% normal goat serum) for 1 hour at room temperature, then with primary antibodies in blocking solution (overnight, 4 °C) and secondary antibodies (1 hour, room temperature). Images were acquired as 12-bit grayscale and prepared using Adobe Photoshop CS5. For quantification, sets of cells were stained simultaneously and imaged with identical settings. Further details are described in Supplementary Methods.

### Cell surface binding assay

For testing for binding of biotin-Aβ_42_ oligomers, COS-7 cells on coverslips were transfected with the indicated expression vectors and maintained for 24 hours. The transfected cells were washed with extracellular solution (ECS) containing 168 mM NaCl, 2.4 mM KCl, 20 mM HEPES (pH 7.4), 10 mM D-glucose, 2 mM CaCl_2_, and 1.3 mM MgCl_2_ with 100 μg/ml BSA (ECS/BSA) and then incubated with ECS/BSA containing 250 nM biotin-Aβ_42_ oligomers (monomer equivalent) for 1 hour at 4 °C to prevent endocytosis. The cells were washed in ECS, fixed with parafix solution for 12 min at room temperature, incubated with blocking solution for 1 hour at room temperature, followed by the immunolabeling of surface HA as described above, and then incubated with Alexa594-conjugated streptavidin (1:4000; Jackson ImmunoResearch) and Alexa488-conjugated anti-rabbit IgG (H+L) (1:500; Invitrogen) for 1 hour at room temperature to label bound biotin-Aβ_42_ oligomers and surface HA, respectively. Further details are described in Supplementary Methods.

### Pull-down assays

Purified soluble recombinant human NRX1βS4(−) ectodomain fused to human Fc (NRX1βS4(−)-Fc, 5268-NX-050, R&D systems) or human Fc (a negative control) generated from the pc4-sp-Fc vector^23^ were used for the pull-down assays. NRX1β-Fc or Fc proteins were pre-immobilized with Protein G magnetic beads (Dynabeads Protein G, Life Technology) in 20 mM sodium phosphate buffer (pH 7.0) for 2 hours at 4 °C. The pre-immobilized NRX1β-Fc or Fc proteins were then incubated with untagged Aβ oligomers in binding solution (20 mM HEPES (pH 7.4), 2 mM CaCl_2_, and 1.3 mM MgCl_2_) for 1 hour at 4 °C. Subsequently, the bead suspensions were washed five times with binding solution. Bound peptides and proteins were eluted with 100 mM glycine-HCl. Eluted samples were diluted in SDS sample buffer without boiling, separated on a 4–20% gradient SDS-PAGE gel and analyzed by western blotting with anti-β-Amyloid 1–16 (1:5000; mouse IgG1; clone 6E10; Covance) or horseradish peroxidase (HRP)-conjugated anti-human Fc (1:10,000; Jackson ImmunoResearch) antibodies.

### Time-lapse imaging

For time-lapse imaging, hippocampal neurons cultured on 18-mm coverslips were cotransfected with a SEP-NRX construct and mCherry at 10 days *in vitro* (DIV) and used for imaging at 20–22 DIV. During imaging, the live transfected neurons were mounted in a Chamlide CMB magnetic chamber (Live Cell Instrument) and maintained in ECS at 37 °C controlled by a Tempcontrol 37–2 device (Pecon Germany) without perfusion. Aβ_42_ oligomers (500 nM, monomer equivalent) were manually added into ECS in the chamber 5 minutes after taking the first image. Fluorescent imaging was performed using a Leica DMIRE2 inverted microscope (Leica Germany) equipped with an Orca ER CCD camera (Hamamatsu Japan) and a 63 × 1.4 NA oil objective lens. All images were acquired by Volocity software (Perkin Elmer) at 1344 × 1024 resolution with 12 bits/pixel.

### Fluorescence quantification

All imaging and image analysis were done while blind to the experimental condition. Analysis was performed by using Metamorph 7.8 software (Molecular Devices), Microsoft Excel, and GraphPad Prism 6. For binding of biotin-Aβ_42_ oligomers and Fc-fusion proteins, the average intensity of bound protein per COS-7 cell area minus off-cell background was normalized to the average intensity of the surface HA signal on COS-7 cells expressing the indicated HA-tagged proteins. For cocultures, fields for imaging were chosen using only the HA and phase contrast channels to locate HA-positive HEK293 cells in neurite-rich regions. The VGLUT1 or VGAT channel was thresholded and the total intensity of the puncta within HA-positive HEK293 cell regions was measured. For time-lapse imaging, the average background intensity of the image before Aβ treatment was measured, and this value was subtracted from the intensity of each frame of the time-lapse image sequences. The axons of transfected neurons were defined based on the morphology of mCherry-expressing neurons. In the image before Aβ treatment, the areas corresponding to puncta of SEP-NRX1β in mCherry-positive axons were manually traced as regions of interest (ROIs) using Metamorph 7.8. The average intensity of SEP and mCherry signals in these ROIs in each frame was measured. To quantify the effects of Aβ treatment on NRX surface expression, the SEP signal was normalized to the mCherry signal. Correction of the image shift in the x–y plane was done by comparing mCherry and SEP images. Pseudo-color images were created based on the fluorescence intensity range of the image prior to the Aβ treatment by Metamorph 7.8.

### Synaptosome preparation and Immunoblotting

Preparation of synaptosome fractions from mice was performed essentially as described previously^26^. For all samples, protein concentrations were measured in DC Protein Assays (Biorad). After normalizing protein concentration, samples were run on 10% polyacrylamide gels. For immunoblotting NRXs, unboiled samples were used. Signals were developed using Immobilon Western Chemiluminescent HRP Substrate (Millipore) and captured by an ImageQuant LAS 4000 instrument (GE healthcare). Band signal intensity was measured using Metamorph 7.8 software and normalized to β-actin signal intensity for quantification. Further details are described in Supplementary Methods.

### Statistical analysis

Statistical tests were performed using GraphPad Prism 6. Data distribution was assumed to be normal. Statistical comparisons were done by Student’s unpaired t test, one-way ANOVA and two-way repeated measures ANOVA with *post hoc* Bonferroni multiple comparisons tests, as indicated in the figure legends. All data are represented as the mean ± standard error of the mean (SEM) from three independent experiments and statistical significance was defined as *P* < 0.05.

## Additional Information

**How to cite this article**: Naito, Y. *et al*. Amyloid-β Oligomers Interact with Neurexin and Diminish Neurexin-mediated Excitatory Presynaptic Organization. *Sci. Rep.* **7**, 42548; doi: 10.1038/srep42548 (2017).

**Publisher's note:** Springer Nature remains neutral with regard to jurisdictional claims in published maps and institutional affiliations.

## Supplementary Material

Supplementary Information
