# Supplementary material for: Amyloid-β Oligomers Interact with Neurexin and Diminish Neurexin-mediated Excitatory Presynaptic Organization
Source: Sci Rep. 2017 Feb 13;7:42548. doi: 10.1038/srep42548 (PMC5304201; doi:10.1038/srep42548)
Supplement: Supplementary Information [file srep42548-s1.pdf]

Supplementary Information

Amyloid-β Oligomers Interact with Neurexin and Diminish Neurexin-mediated Excitatory Presynaptic Organization

Yusuke Naito[1,2], Yuko Tanabe[1], Alfred Kihoon Lee[1,2], Edith Hamel[3] & Hideto Takahashi[1,4*]

1. Synapse Development and Plasticity Research Unit, Institut de recherches cliniques de Montréal, Montréal, Québec, H2W 1R7, Canada
2. Integrated Program in Neuroscience, McGill University, Montréal, Québec, H3A 2B4, Canada
3. Laboratory of Cerebrovascular Research, Montreal Neurological Institute, McGill University, Montréal, Québec, H3A 2B4, Canada
4. Department of Medicine, Université de Montréal, Montréal, Québec, Canada, H3T 1J4; Division of Experimental Medicine, McGill University, Montréal, Québec, H3A 0G4, Canada

*Corresponding authors:
Hideto Takahashi: Institut de Recherches Cliniques de Montréal, 110 avenue des Pins Ouest, Montréal, Québec, H2W 1R7, Canada. E-mail: Hideto.Takahashi@ircm.qc.ca

Supplementary Figures

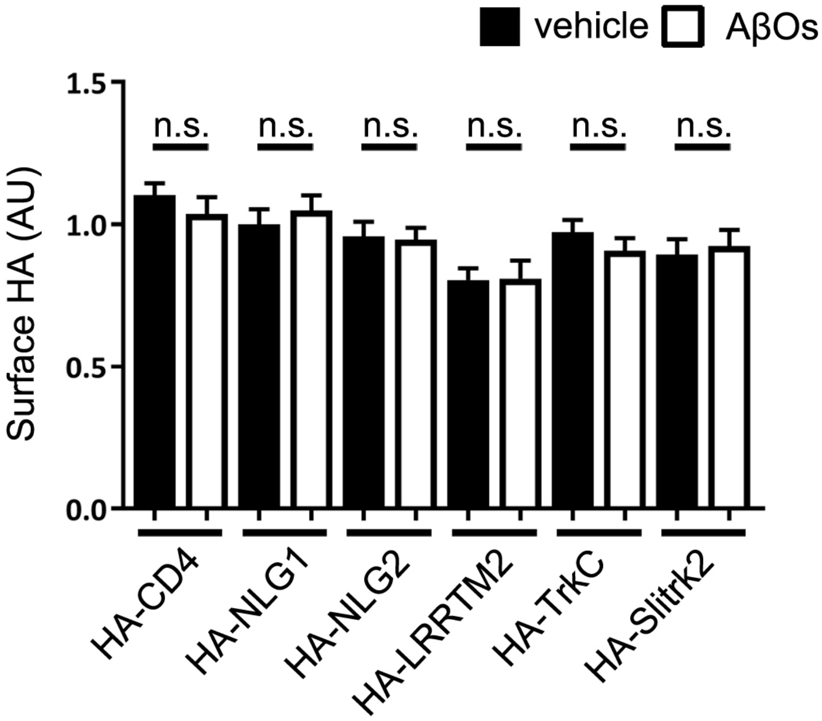

Supplementary Figure 1. Aβ_{42} oligomers have no effect on surface expression of the tested synaptic organizers expressed on HEK cells in artificial synapse formation assays.

Quantification of the average intensity of surface HA of the indicated extracellularly HA-tagged proteins expressed on HEK293 cells cocultured with cultured hippocampal neurons and treated with Aβ_{42} oligomers (AβOs, 500 nM, monomer equivalent) or vehicle. *n* = 30 cells for each construct from three independent experiments, one-way ANOVA, $P < 0.001$. n.s., not significant by Bonferroni multiple comparisons test. Data are presented as mean ± SEM.

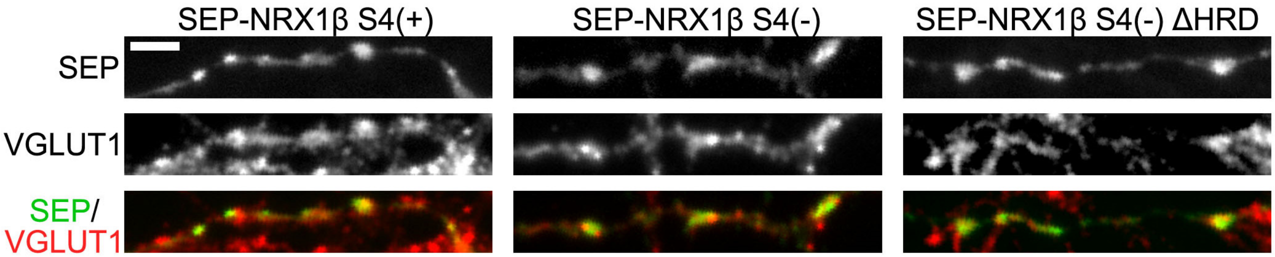

Supplementary Figure 2. SEP-NRX1β puncta colocalize with VGLUT1 puncta

Representative images of cultured hippocampal neurons expressing extracellularly super-ecliptic pHluorin (SEP)-tagged NRX1βS4(+), SEP-tagged NRX1βS4(-) or SEP-tagged NRX1βS4(-) lacking its histidine-rich domain (SEP-NRX1βS4(-) ΔHRD), immunostained for excitatory presynaptic vesicle marker VGLUT1. Note that each SEP-NRX1β construct displays similar punctate distribution on axons and that these puncta nicely colocalize with VGLUT1 puncta. Scale bar represents 5 μm.

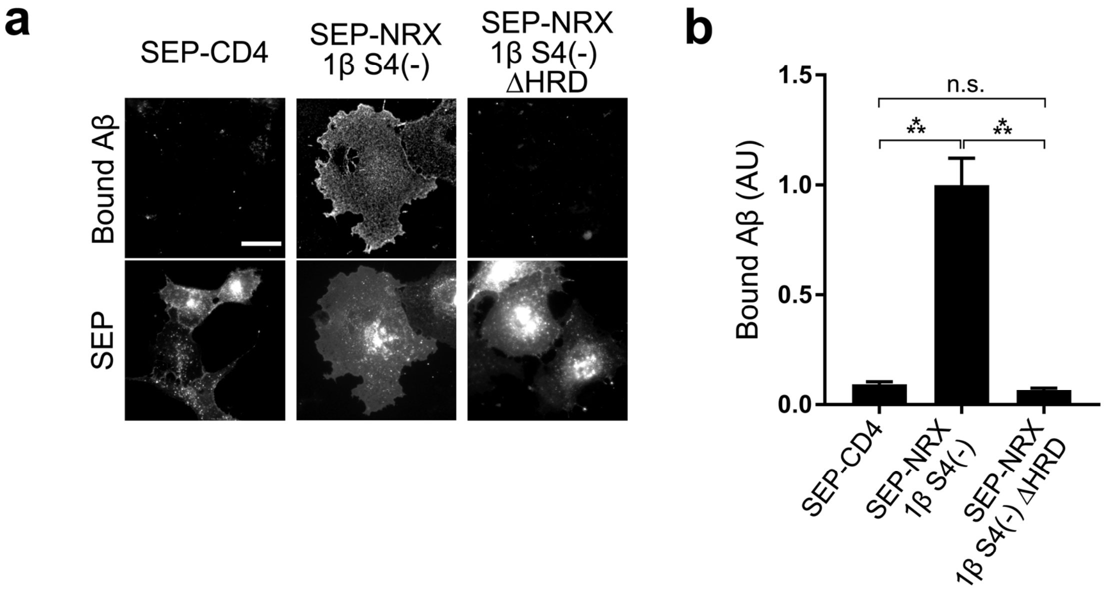

Supplementary Figure 3. Aβ_{42} oligomers do not bind to SEP-NRX1βS4(-)ΔHRD

(**a**) Representative images showing the binding of biotin-Aβ_{42} oligomers (250 nM, monomer equivalent) to COS-7 cells expressing super-ecliptic pHluorin (SEP) tagged-CD4 (a negative control), SEP-tagged NRX1βS4(-) or SEP-tagged NRX1βS4(-) lacking the histidine-rich domain (SEP-NRX1βS4(-) ΔHRD). Note that cells expressing SEP-NRX1βS4(-) ΔHRD have no signal of bound Aβ.

(**b**) Quantification of bound biotin-Aβ_{42} oligomers for each SEP-NRX1β construct. n = 30 cells for each construct from three independent experiments, one-way ANOVA, $P < 0.0001$, ***$P \leq 0.0001$ and n.s., not significant by Bonferroni multiple comparisons tests.

Scale bar represents 30 μm .Data are presented as mean ± SEM.

Figure 1a

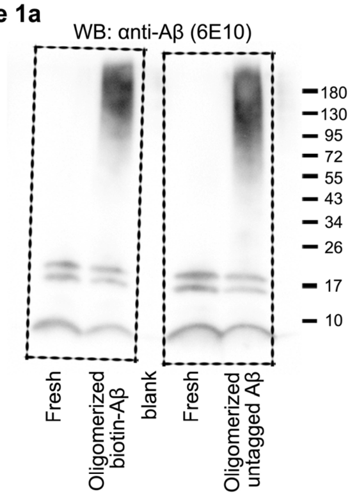

Figure 7a

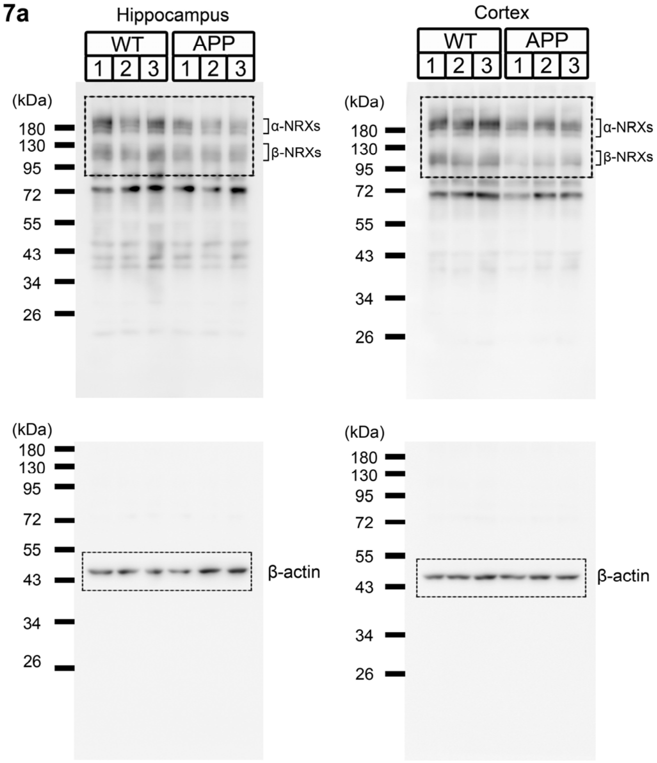

Figure 2a

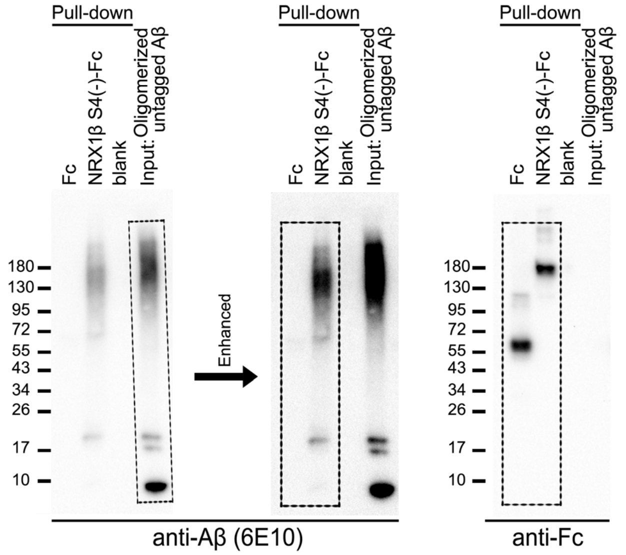

Supplementary Figure 4. Uncropped full-length images of immunoblots in Figure 1a, Figure 2a and Figure 7a

Dot line boxes indicate the cropped images used in each figure.

Supplementary Methods

Plasmids

To generate a series of extracellularly HA-tagged neurexin (HA-NRX) constructs, cDNA encoding the mature form of each NRX isoform was subcloned into spNRX1β-HA-C1, a vector containing a CMV promoter upstream of the N-terminal signal sequence of NRX1β (spNRX1β) followed by HA and a multiple cloning site. The following NRX vectors were used as a PCR template for the subcloning: intracellular CFP-tagged mouse NRX1βS4(+), 1βS4(-), 1αS4(+), 1αS4(-), 2αS4(+), 2αS4(-), 3αS4(+), and 3αS4(-) (kindly provided by Dr. Ann Marie Craig (University of British Columbia)) and intracellular V5-tagged mouse NRX2βS4(+), 2βS4(-), 3βS4(+), and 3βS4(-) (kindly provided by Dr. Takeshi Uemura (Shinshu University)). For extracellularly HA-tagged PirB (HA-PirB) and prion protein (HA-PrP^c), the coding sequences for the mature forms of mouse PirB (amino acid (aa) 25-841) and mouse PrP^c (aa 23-254) were amplified by PCR from mouse PirB-ires-GFP and GFP-mouse PrP^c, respectively, and then subcloned into spNRX1β-HA-C1. For extracellularly HA-tagged LRRTM2 (HA-LRRTM2), the coding sequence for the mature form of rat LRRTM2 (aa 34-515) was amplified by PCR from rat LRRTM2-CFP and then subcloned into spTrkC-HA-C1[1]. The following plasmids were kind gifts: PirB-ires-GFP from Dr. Carla Shatz (Stanford University), GFP-PrP^c from Dr. Marco Prado (The University of Western Ontario), HA-NLG1A(+)B(+), HA-NLG1A(+)B(-), HA-NLG1A(-)B(+), HA-NLG1A(-)B(-), NRX1βS4(-)-Fc from Dr. Peter Scheiffele (University of Basel) via Addgene, HA-NLG2 and YFP-NLG3, LRRTM2-CFP from Dr. Ann Marie Craig, LAR-CFP from Dr. Eunjoon Kim (Korea Advanced Institute of Science and Technology), HA-GluRδ1 and HA-GluRδ2 from Dr. Michisuke Yuzaki (Keio University), IL1RAPL1-pFLAG and IL1RAcP-pFLAG from Dr. Tomoyuki Yoshida (Toyama University). The other constructs used in the Aβ-binding screen and coculture assays were described previously[1,2]. For β-NRX constructs lacking their N-terminal histidine-rich domain (HRD), the coding sequence for the mature forms of NRX1β lacking HRD (aa 50-83), NRX2β lacking HRD (aa 54-87) and NRX3β lacking HRD (aa 48-81) were subcloned into spNRX1β-HA-C1 following the NRX1β signal sequence and HA. The construct expressing LRRTM2 ectodomain fused to human Fc (pc4-LRRTM2-Fc) was generated by

subcloning the coding sequences for the LRRTM2 ectodomain (aa 1-421), into pc4-sp-Fc[1,2]. For the construct expressing the NLG1 ectodomain fused to Fc (pc4-NLG1-Fc), the coding sequence for the mature form of NLG1A(-)B(-) (aa 57-675) was subcloned into pc4-sp-Fc, following spNRX1β. For extracellularly super-ecliptic pHluorin (SEP)-tagged neurexin1β (SEP-NRX1β) constructs, the coding sequence for the mature form of each NRX1β was subcloned into spNRX1β-SEP-C1, a vector containing a CMV promoter upstream of spNRX1β followed by the SEP coding region and a multiple cloning site. All constructs were verified by DNA sequencing.

Animals

All animal experiments were carried out in accordance with the Canadian Council on Animal Care guidelines and approved by the IRCM Animal Care Committee and the McGill University Animal Care Committee. We used heterozygous transgenic adult C57BL/6 mice (6 months old, mixed sex) expressing the human amyloid precursor protein (hAPP) carrying the Swedish (K670N, M671L) and Indiana (V717F) familial AD mutations driven by the platelet-derived growth factor (PDGF) β-chain promoter (APP mice, J20 line)[3] and age-matched wild-type (WT) littermates.

Preparation of Aβ_{42} oligomers

Aβ(1–42) (r-peptide, A-1002-2, 1 mg) and biotin-tagged Aβ(1–42) (Anaspec, AS-23523-05, 0.5 mg) were used to generate oligomeric forms essentially as described previously[4]. Briefly, lyophilized peptides were dissolved in 1,1,1,3,3,3-hexafluoro-2-propanol (HFIP; Sigma-Aldrich, Cat # 52517) to ensure that the starting material was in a homogenous non-aggregated monomeric state, then aliquots containing peptide were placed in low-binding polypropylene microcentrifuge tubes for 2 hours at room temperature for peptide monomerization. The HFIP was evaporated in a vacuum centrifuge concentrator (SPD131, Thermo Scientific) and the resulting Aβ peptide films were stored at −80°C with desiccant. Prior to use, each peptide film was reconstituted in dimethylsulfoxide (DMSO, Sigma-Aldrich, Hybri-Max D-2650) to obtain a 1 mM Aβ stock solution, which was then incubated in a bath sonicator for 10 minutes. The

peptide stock was then diluted to a concentration of 100 μM with 10 mM Tris-HCl, pH 7.4, and incubated for 48 hours at 22°C to facilitate the formation of higher molecular weight oligomers. These preparations were stored at −80°C or used in experiments immediately. Individual Aβ oligomer stocks were never thawed and re-frozen. To confirm oligomer formation, the preparation was run on a 4-20% TGX precast gel (Biorad) and immunoblotted with anti-β-Amyloid 1-16 (1:5000; mouse IgG1; clone 6E10; Covance).

Neuron culture, coculture-based artificial synapse formation assay and immunocytochemistry

Cultures of rat hippocampal neurons, COS-7 cells, HEK293 cells, coculture-based artificial synapse formation assays, and immunocytochemistry were performed essentially as reported previously[1,2]. Transfections into COS-7 and HEK293 cells were performed using TransIT-LT1 (Mirus Bio. LLC). For transfections into hippocampal neurons, the ProFection Mammalian Transfection System (Promega) was used. For artificial synapse formation assays, transfected HEK293 cells were co-cultured with rat hippocampal neurons. Cultures were fixed with parafix solution (4% paraformaldehyde and 4% sucrose in PBS (pH 7.4)) for 12 minutes followed by permeabilization with PBST (PBS + 0.2% Triton X-100). They were incubated with blocking solution (PBS + 3% bovine serum albumin (BSA) and 5% normal goat serum) for 1 hour at room temperature, then with primary antibodies in blocking solution (overnight, 4°C) and secondary antibodies (1 hour, room temperature). The following primary antibodies were used for immunocytochemistry: anti-VGLUT1 (1:1,000; guinea pig, AB5905, Millipore), anti-VGAT (1:1,000; rabbit, 131 003, Synaptic Systems), anti-HA (1:1,000; mouse IgG2b, 12CA5, Roche), anti-HA (1:2,000; rabbit IgG, ab9110, Abcam), and anti-Flag (1:2000; mouse IgG1, M2, Sigma). Highly cross-adsorbed, Alexa-dye conjugated secondary antibodies generated in goat towards the appropriate species were used for detection (1:1000; Alexa-488, Alexa-568, and Alexa-647; Invitrogen). To label surface HA, cultures were fixed in parafix solution, blocked without permeabilization, and then incubated with anti-HA antibody at 4°C overnight. Fluorescent images were captured on a Leica DM6000 fluorescent microscope with a 40X 0.75 NA air objective or a 63X 1.4 NA oil objective and a Hamamatsu cooled CCD camera using Volocity software (Perkin Elmer). Images were acquired as 12-bit grayscale and

prepared using Adobe Photoshop CS5. For quantification, sets of cells were stained simultaneously and imaged with identical settings.

Cell surface binding assay

For testing for binding of biotin-Aβ_{42} oligomers, COS-7 cells on coverslips were transfected with the indicated expression vectors and maintained for 24 hours. The transfected cells were washed with extracellular solution (ECS) containing 168 mM NaCl, 2.4 mM KCl, 20 mM HEPES (pH 7.4), 10 mM D-glucose, 2mM $CaCl_2$, and 1.3mM $MgCl_2$ with 100 µg/ml BSA (ECS/BSA) and then incubated with ECS/BSA containing 250 nM biotin-Aβ_{42} oligomers (monomer equivalent) for 1 hour at 4°C to prevent endocytosis. The cells were washed in ECS, fixed with parafix solution for 12 min at room temperature, incubated with blocking solution for 1 hour at room temperature, followed by the immunolabeling of surface HA as described above, and then incubated with Alexa594-conjugated streptavidin (1:4000; Jackson ImmunoResearch) and Alexa488-conjugated anti-rabbit IgG (H+L) (1:500; Invitrogen) for 1 hour at room temperature to label bound biotin-Aβ_{42} oligomers and surface HA, respectively. To test the effects of Aβ_{42} oligomers on NRX-NLG1 and NRX-LRRTM2 interactions, COS-7 cells expressing HA-NRX1β constructs were incubated with ECS/BSA containing 20 nM NLG1-Fc or 20 nM LRRTM2-Fc proteins with or without 500 nM biotin-Aβ_{42} oligomers (monomer equivalent) for 1 hour at 4°C. NLG1-Fc and LRRTM2-Fc proteins were generated from pc4-NLG1-Fc and pc4-LRRTM2-Fc vectors, respectively, as essentially described before[1,2]. Bound Fc proteins were labeled with Alexa594-conjugated donkey anti-human IgG (H+L) (1:500; Jackson ImmunoResearch).

Pull-down assays

Purified soluble recombinant human NRX1βS4(-) ectodomain fused to human Fc (NRX1βS4(-)-Fc, 5268-NX-050, R&D systems) or human Fc (a negative control) generated from the pc4-sp-Fc vector[1] were used for the pull-down assays. NRX1β-Fc or Fc proteins were pre-immobilized with Protein G magnetic beads (Dynabeads Protein G, Life Technology) in 20 mM sodium phosphate buffer (pH 7.0) for

2 hours at 4°C. The pre-immobilized NRX1β-Fc or Fc proteins were then incubated with untagged Aβ oligomers in binding solution (20 mM HEPES (pH 7.4), 2mM $CaCl_2$, and 1.3mM $MgCl_2$) for 1 hour at 4°C. Subsequently, the bead suspensions were washed five times with binding solution. Bound peptides and proteins were eluted with 100 mM glycine-HCl. Eluted samples were diluted in SDS sample buffer without boiling, separated on a 4-20% gradient SDS-PAGE gel and analyzed by western blotting with anti-β-Amyloid 1-16 (1:5000; mouse IgG1; clone 6E10; Covance) or horseradish peroxidase (HRP)-conjugated anti-human Fc (1:10,000; Jackson ImmunoResearch) antibodies.

Time-lapse imaging

For time-lapse imaging, hippocampal neurons cultured on 18-mm coverslips were cotransfected with a SEP-NRX construct and mCherry at 10 days *in vitro* (DIV) and used for imaging at 20-22 DIV. During imaging, the live transfected neurons were mounted in a Chamlide CMB magnetic chamber (Live Cell Instrument) and maintained in ECS at 37°C controlled by a Tempcontrol 37-2 device (Pecon Germany) without perfusion. $A\beta_{42}$ oligomers (500 nM, monomer equivalent) were manually added into ECS in the chamber 5 minutes after taking the first image. Fluorescent imaging was performed using a Leica DMIRE2 inverted microscope (Leica Germany) equipped with an Orca ER CCD camera (Hamamatsu Japan) and a 63X 1.4 NA oil objective lens. All images were acquired by Volocity software (Perkin Elmer) at 1344 × 1024 resolution with 12 bits/pixel.

Fluorescence quantification

All imaging and image analysis were done while blind to the experimental condition. Analysis was performed by using Metamorph 7.8 software (Molecular Devices), Microsoft Excel, and GraphPad Prism 6. For binding of biotin-$A\beta_{42}$ oligomers and Fc-fusion proteins, the average intensity of bound protein per COS-7 cell area minus off-cell background was normalized to the average intensity of the surface HA signal on COS-7 cells expressing the indicated HA-tagged proteins. For cocultures, fields for imaging were chosen using only the HA and phase contrast channels to locate HA-positive HEK293 cells in

neurite-rich regions. The VGLUT1 or VGAT channel was thresholded and the total intensity of the puncta within HA-positive HEK293 cell regions was measured. For time-lapse imaging, the average background intensity of the image before Aβ treatment was measured, and this value was subtracted from the intensity of each frame of the time-lapse image sequences. The axons of transfected neurons were defined based on the morphology of mCherry-expressing neurons. In the image before Aβ treatment, the areas corresponding to puncta of SEP-NRX1β in mCherry-positive axons were manually traced as regions of interest (ROIs) using Metamorph 7.8. The average intensity of SEP and mCherry signals in these ROIs in each frame was measured. To quantify the effects of Aβ treatment on NRX surface expression, the SEP signal was normalized to the mCherry signal. Correction of the image shift in the x–y plane was done by comparing mCherry and SEP images. Pseudo-color images were created based on the fluorescence intensity range of the image prior to the Aβ treatment by Metamorph 7.8.

Synaptosome preparation

Preparation of synaptosome fractions from mice was performed essentially as described previously[5]. All steps were performed at 4°C. The cerebral cortex of each mouse was homogenized in 2 mL of Buffer A (5 mM HEPES, pH 7.4, 1 mM $MgCl_2$, 0.5 mM $CaCl_2$, 1 mM DTT, 0.32 M sucrose, supplemented with protease inhibitors) by passing the lysate 9 times through a 1-mL syringe without needle and then 5 times through a 1-mL syringe with a 18-gauge needle. The hippocampi from each two mice were homogenized together in 1 mL of Buffer A by passing the lysate as described above to obtain enough material as a single sample. The suspension was centrifuged for 10 minutes at 1,400 g, and the supernatant was set aside. The pellet was resuspended in Buffer A (2 mL in cortex and 1 mL in hippocampus) using a 1-mL syringe with a 23-gauge needle 3-5 times, and the suspension was centrifuged for 10 minutes at 750-1,000 g. The supernatant was pooled with the supernatant collected after the first centrifugation (fraction: S1). The pooled fractions were centrifuged for 10 minutes at 12,000 g, and the supernatant was removed (fraction, Cytoplasm). The pellet was resuspended in 1 mL (cortex) or 0.5 mL (hippocampus) of Buffer B (6 mM Tris, pH 8.1, 0.32 M sucrose, 1 mM EDTA, 1 mM EGTA, 1

mM DTT, supplemented with protease inhibitors) (fraction, P2). Fraction P2 was resuspended in Buffer B and centrifuged for 15 minutes at 14,000 g. The pellet was resuspended in Buffer B (1 mL in cortex and 0.5 mL in hippocampus) (fraction, P2'). A sucrose gradient was prepared with (bottom to top) 1.2 M, 1.0 M and 0.85 M sucrose in 6 mM Tris, pH 8.1. The P2' fraction was layered over the sucrose gradient and centrifuged for 2 hours at 82,500 g using a SW55Ti rotor (Beckman) in case of cortex or a TLS55 rotor (Beckman) in case of hippocampus. Material at the interface between the 1.0 M and 1.2 M sucrose layers was collected (fraction: synaptosome).

Immunoblotting

For all samples, protein concentrations were measured in DC Protein Assays (Biorad). After normalizing protein concentration, samples were run on 10% polyacrylamide gels. For immunoblotting NRXs, unboiled samples were used. Gels were transferred onto Immobilon P membranes (Millipore). Membranes were blocked in 5% skim milk and 0.1% Triton X-100 in PBS and incubated with one of the following primary antibodies: anti-NRX1/2/3 (1:1,000; rabbit, 175 003, Synaptic Systems) and anti-β-actin (1:2,000; rabbit, ab8227, Abcam). Membranes were washed with PBS containing 0.1% Triton X-100 and incubated with HRP-conjugated anti-rabbit IgG (H+L) (1:10,000; 111-035-144, Jackson ImmunoResearch). Signals were developed using Immobilon Western Chemiluminescent HRP Substrate (Millipore) and captured by an ImageQuant LAS 4000 instrument (GE healthcare). Band signal intensity was measured using Metamorph 7.8 software and normalized to β-actin signal intensity for quantification.

Statistical analysis

Statistical tests were performed using GraphPad Prism 6. Data distribution was assumed to be normal. Statistical comparisons were done by Student's unpaired t test, one-way ANOVA and two-way repeated measures ANOVA with *post hoc* Bonferroni multiple comparisons tests, as indicated in the figure

legends. All data are represented as the mean ± standard error of the mean (SEM) from three independent experiments and statistical significance was defined as $P < 0.05$.

References

1. Takahashi, H., *et al.* Postsynaptic TrkC and presynaptic PTPsigma function as a bidirectional excitatory synaptic organizing complex. *Neuron* **69**, 287-303 (2011).

2. Takahashi, H., *et al.* Selective control of inhibitory synapse development by Slitrk3-PTPdelta trans-synaptic interaction. *Nat Neurosci* **15**, 389-398, S381-382 (2012).

3. Mucke, L., *et al.* High-level neuronal expression of abeta 1-42 in wild-type human amyloid protein precursor transgenic mice: synaptotoxicity without plaque formation. *J Neurosci* **20**, 4050-4058 (2000).

4. Caetano, F.A., *et al.* Amyloid-beta oligomers increase the localization of prion protein at the cell surface. *J Neurochem* **117**, 538-553 (2011).

5. Linhoff, M.W., *et al.* An unbiased expression screen for synaptogenic proteins identifies the LRRTM protein family as synaptic organizers. *Neuron* **61**, 734-749 (2009).
